# Oral Mycobiome and COVID-19

**DOI:** 10.3390/microorganisms11040982

**Published:** 2023-04-10

**Authors:** Francesco D’Ambrosio

**Affiliations:** Department of Medicine, Surgery and Dentistry, University of Salerno, 84084 Salerno, Italy; fradambrosio@unisa.it or francesco87fd@libero.it

The most common signs and symptoms of COVID-19 include fever, cough, dyspnea, conjunctivitis, diarrhea, and olfactory and gustatory disturbances [1]. Several authors have also described oral manifestations that are likely related to the viral infection itself or to treatment or disease complications [1,2], including oral candidiasis [3].

Indeed, it has been hypothesized that the immunocompromised state of SARS-CoV-2-positive patients predisposes them to the development of fungal and bacterial infections. Moreover, critically ill patients who required mechanical ventilation and were admitted to the intensive care unit (ICU) [4] frequently experienced additional complications, such as healthcare-associated infections (HAIs), that are related to long hospital stays and overuse or misuse of drugs commonly used to treat COVID-19, such as antibiotics and corticosteroids [5,6,7,8]. Specifically, SARS-CoV-2-positive patients in the ICU were ten times more likely to develop secondary fungal or bacterial HAIs than viral coinfections [5].

Fungal and bacterial coinfections have been estimated to increase mortality and morbidity in COVID-19 patients, especially if they have acute respiratory distress syndrome (ARDS) [9]. As a counterpart, Candida colonization has been associated with an increased risk of developing ARDS in SARS-CoV-2-positive individuals [10].

Specifically, bacterial coinfections ranged from 2.5% to 5.1% in SARS-CoV-2-positive patients in ICUs, compared with less than 1% rates in non-hospitalized patients [11]. After *Klebsiella pneumonia* (10.9%) and *Acinetobacter baumanii* (8.8%), *Candida* was the third most common pathogen associated with systemic secondary infections in COVID-19 patients (4.1%), in India [10]. In critically ill COVID-19 patients, fungal superinfections are mostly sustained by *Aspergillus* and *Candida*, although mucormycosis also seems to be a growing concern [4].

Especially in immunocompromised COVID-19 patients in ICUs, *Candida* may evolve from a normal commensal of the skin, mucous membranes, respiratory tract, digestive tract, and urinary tract to an opportunistic pathogen [12]. *Candida* pathogenicity can be promoted via immunosuppression, the prolonged use of drugs, hyposalivation, direct cell damage by SARS-CoV-2, and mechanical ventilation. Indeed, mechanical ventilators are usually associated with an increased risk of ventilator-associated pneumonia, which is associated with microorganisms from the oropharynx and oral cavity [5].

Based on a body of evidence, the existence of an oral–intestinal–pulmonary microbiome axis has been previously suggested [13]. Individuals with chronic obstructive pulmonary diseases often have intestinal hyperpermeability and a higher incidence of inflammatory bowel disease [14]. At the same time, respiratory infections are often accompanied by gastrointestinal symptoms or dysfunction, due to the altered gut microbiome, which indirectly regulates the macrophage response to respiratory pathogens [13,15]. In fact, immune cells from the gut diffuse into the systemic circulation and reach the respiratory tract, allowing the host to fight infections [13].

In addition, the oral microbiota is linked to the respiratory and gastrointestinal tracts through anatomical contiguity. Healthy humans frequently harbor oral anaerobic microorganisms such as *Prevotella* and *Veillonella* spp., probably due to continuous microaspiration through contiguity [16]. It is well known that some systemic conditions and diseases, such as cardiovascular disease, obesity, diabetes, or preterm birth, contribute to the onset and exacerbation of periodontitis [17,18]. On the other hand, it is also known that oral and, specifically, periodontal microorganisms can exacerbate or promote the onset of systemic diseases through their metabolites, contiguity diffusion, or modulation of host immune responses [19].

In SARS-CoV-2-positive subjects with oropharyngeal candidiasis, *Candida albicans* spp. was most frequently detected (70.7%), followed by *Candida glabrata* (10.7%), *Candida dublinien sis* (9.2%), *Candida parapsilosis sensu stricto* (4.6%), *Candida tropicalis* (3%), and *Candida krusei* (1.5%) [5].

*Candida* spp. can spread systemically through the blood; when candidaemia occurs, significant morbidity rates, ranging from 71% to 79%, have generally been reported [5]. Notably, *Candida* spp. from the oral cavity can be passively transported to the upper and lower airways via intubation beyond mechanical ventilation devices and can translocate to the esophagus and upper gastrointestinal tract [20].

Thus, *Candida* spp. may, in turn, influence the lung infection caused by SARS-CoV-2 or even alter the gastrointestinal microbiome, which in turn can induce local and systemic damage. Correspondingly, Zuo et al. showed the enrichment of *C. albicans*, *Candida auris*, and *Aspergillus flavus* in the intestinal microbiome in SARS-CoV-2-positive subjects [21], and the gut colonization of *Candida albicans* in mice aggravated both intestinal diseases, with allergic diarrhea, and non-intestinal ones, by inducing limb joint inflammation in collagen-induced arthritis [22].

Consequently, even though SARS-CoV-2-positive patients in ICUs often require other non-deferrable interventions [23], the occurrence of oral candidiasis in these patients should not be underestimated, especially because bacterial and fungal coinfections have been associated with approximately 50% of deaths due to COVID-19 [4].

Furthermore, the prolonged use of medications during hospitalization and drug misuse or non-adherence to domiciliary therapy are the factors involved in developing microorganism resistance to antimicrobial drugs [24,25]. Although reported in patients with recurrent infections or human immunodeficiency virus infection, resistance to antimycotics in *C. albicans* infection is rare [26]. However, some non-albicans *Candida* species, such as *C. krusei*, are inherently resistant or less sensitive to different classes of antimycotics [26]. In addition, other species, such as *C. glabrata*, may develop acquired resistance following exposure to antimycotics [26]. Fortunately, multidrug resistance remains uncommon, despite being increasingly described [26]. 

Nevertheless, compared with other *Candida* spp. mostly associated with sporadic infections, a greater capacity for nosocomial transmission was shown by *C. auris* [27]. Moreover, even before the outbreak of the COVID-19 pandemic, researchers around the world revealed that *C. auris* developed a level of multidrug resistance not observed in other *Candida* species [10,27].

Therefore, subjects with COVID-19 presenting with oral candidiasis, especially those with severe illness in ICUs, should be accurately managed with a multidisciplinary approach [28] to reduce the risk of inaccurate and untimely diagnosis and treatment and prevent the consequent possible development of further resistant mycotic strains, especially in the hospital setting.

## Data Availability

All data generated or analyzed during this study are included in this article.
